# A Composite of Hydrogel Alginate/PVA/r-GO for Scaffold Applications with Enhanced Degradation and Biocompatibility Properties

**DOI:** 10.3390/polym15030534

**Published:** 2023-01-19

**Authors:** Amaliya Rasyida, Salma Halimah, Ika Dewi Wijayanti, Sigit Tri Wicaksono, Haniffudin Nurdiansah, Yohannes Marudut Tua Silaen, Yatim Lailun Ni’mah, Hosta Ardhyananta, Agung Purniawan

**Affiliations:** 1 Department of Materials and Metallurgical Engineering, Faculty of Industrial Technology and System Engineering, Institut Teknologi Sepuluh Nopember, Surabaya 60111, Indonesia; 2 Department of Mechanical Engineering, Faculty of Industrial Technology and System Engineering, Institut Teknologi Sepuluh Nopember, Surabaya 60111, Indonesia; 3 Department of Chemistry, Faculty of Sains and Analytica Data, Institut Teknologi Sepuluh Nopember, Surabaya 60111, Indonesia

**Keywords:** Alginate/PVA/r-GO composite, hydrogel, tissue engineering, biocompatibility, degradation

## Abstract

We reported in this study the interrelation between the addition of 0.4, 0.8, 1.2, and 1.6 wt. % reduced graphene oxide (r-GO) into PVA/Alginate and their degradation and biocompatibility properties. The r-GO was synthesized by using the Hummer’s method. A crosslinker CaSO_4_ was added to prepare Alginate/PVA/r-GO Hydrogel composite. A Field Emission in Lens (FEI)-scanning electron microscopy (SEM), along with X-ray energy dispersive spectroscopy (EDS), was performed, characterizing the morphology of the composite. A compressive test was conducted, determining the mechanical properties of the composite with the highest achieved 0.0571 MPa. Furthermore, in vitro cytotoxicity was conducted to determine the biocompatibility properties of the studied composite. An MTT assay was applied to measure cell viability. In general, the presence of r-GO was found to have no significant effect on the morphology of the hydrogel. Indeed, adding 0.4% r-GO to the PVA/Alginate increased the cell viability up to 122.26 ± 0.93, indicating low toxicity. The studied composites have almost no changes in weight and shape, which proves that low degradation occurred in addition to this after 28 days of immersion in saline phosphate buffer solution. In conclusion, achieving minimal degradation and outstanding biocompatibility lead to PVA/Alginate/r-GO hydrogel composites being the most attractive materials for tissue engineering applications.

## 1. Introduction

A multidisciplinary area called tissue engineering was developed to address medical issues such as organ failure and tissue loss. This field entails a fundamental comprehension of the links between the structure and function of normal and abnormal tissues as well as the creation of biological replacements that restore, maintain, or improve tissue function. This area needs three important aspects, namely cells, bioactive molecules, and scaffolds [1,2]. On the other hand, a biomaterial known as hydrogel is employed in biomedical applications such as tissue engineering, drug delivery, wound dressing, and soft tissue electronics owing to its unique properties, such as biocompatibility and the capacity to mimic many characteristics of the natural [2,3,4,5,6,7,8]. Hydrogels are increasingly being used since they can mimic the specific environment of the extracellular matrix and in bioprocess engineering for immobilizing cells or enzymes as catalysts, drug carriers, cartilage and skin substitutes, wound dressings, a scaffold for cell culture, and as an antifouling agent [9,10]. Injectable scaffolds are attractive for tissue regeneration since they have several benefits over pre-formed scaffolds [11]. The use of hydrogel as injectable biomaterials can be easily injected into the area of the body through a needle, thereby minimizing the effect of treatment due to the minimally invasive injury [12].

Hydrogel materials for regenerative applications are usually made from naturally derived or synthetic polymers (such as polyethylene glycol, polyacrylamide, and polyvinyl alcohol). In this case, hydrogels have been used as scaffolds that mimic extracellular matrices, providing structural integrity and bulk for cellular organization and morphogenic guidance, while encapsulating and delivering cells. Hydrogel scaffolds are employed to add bulk and mechanical structures to a tissue construct, whether cells are floating within or adhering to the 3D hydrogel framework [13]. Hydrogels have become one of the essential scaffolds for tissue engineering because of their biocompatibility, biodegradability, and water solubility. Many researchers suggest that alginate could be an alternative to hydrogel in tissue engineering [14,15,16]. One of sodium alginate’s key characteristics is its capacity to create hydrogels, which is mostly owing to the substitution of sodium ions in the guluronic acid residues with various divalent cations (Ca^2+^, Sr^2+^, Ba^2+^, and others) during the manufacturing process. A 3D network is then generated as a result of the divalent cation’s binding to the -L-guluronic block (and between two distinct chains) [16]. 

Alginate is a natural polysaccharide that can be found in algae plants. The utilization of alginate as a biomaterial can be easily physically and chemically modified to obtain the properties, functions, applications, and structures, such as being modified into hydrogels, microspheres, microcapsules, sponges, foams, and fibers. The utilization of alginate as a biomaterial can be easily physically and chemically modified to obtaining the properties, functions, applications, and structures, such as being modified into hydrogels, microspheres, microcapsules, sponges, foams, and fibers. The physical characteristics of the resulting gels can change as the molecular weight of the alginate increases (e.g., high molecular weight alginate solution becomes greatly viscous). Alginate has the ability to create gels by switching out the sodium ions from guluronic acids for divalent cations such as Ca^2+^, which use the “egg-box” concept to crosslink the polymer chains [16]. This modification can increase applications in various desired biomaterial fields [17]. 

Emily et al. [18] observed the behavior of gelation time in alginate material by varying the CaCl_2_ cross-linked alginate with calcium carbonate (CaCO_3_) and glucono-δ-lactone (GDL) based on a previous study of synthesizing hydrogel for tissue engineering applications. This research obtained an appropriate composition for obtaining the right gelation time. In addition, Lu Zhang et al. [9] reported that adding graphene oxide (GO) into the bio composite hydrogel PVA could increase the mechanical properties (tensile stress, elongation break, and compressive strength).

Poly (vinyl alcohol) is a synthetic polymer widely used as a friendly thermoplastic for tissue that is harmless and non-toxic. In addition, poly (vinyl alcohol) is a biodegradable material that can be improved in its degradability through hydrolysis due to the presence of hydroxyl groups on carbon atoms [19,20]. PVA is widely used for hydrogel [21,22] and has been investigated for injectable materials in nucleus pulposus replacement therapy [23]. On the other hand, graphene is a carbon atom composed of monolayers and forms 2D structures. Graphene-based composites can be used for bone repair or regeneration since they can induce osteogenic and chondrogenic stem cell processes. Adding a small amount of graphene can significantly improve the mechanical properties of composites as compared to the other types of reinforcement in composites [24]. This research has the objective of improving the mechanical properties of hydrogel-based alginate, which is further used for injectable scaffolds in tissue engineering. Furthermore, our previous result [25] showed that the hydrogel composite of Alginate/PVA/r-GO has good injectable performance, and the addition of r-GO was found to accelerate the gelling time of the hydrogel composite and lower the swelling ratio. This result can be used as a preliminary result to further characterize the potential use of this hydrogel composite as an injectable material for tissue engineering applications. To the best of our knowledge, no reports have studied the effect of r-GO on its degradation and biocompatibility in Alginate/PVA/r-GO hydrogel composites.

## 2. Experimental Procedure

### 2.1. Composite Synthesis

r-GO was produced using Hummer’s method described by Nurdiansah et al. before synthesizing the hydrogel composite [26]. The r-GO suspension was sonicated until it became powder. 1.5 g of Sodium Alginate was mixed with the r-GO suspension, then stirred for 1 h at 200 rpm. PVA 0.63 gr was dissolved in 15 mL distilled water at a temperature of 120 °C for 1 h at a speed of 200 rpm and continued by mixing the Alginate/r-GO solution with the PVA solution. 0.3 g of Na_2_HPO_4_ was dissolved in 5 mL of distilled water and mixed into Alginate/PVA/r-GO for 1.5 h at 200 rpm. A total of 1.5 g of CaSO_4_ was dissolved into 10 mL of distilled water and added to the prior solution for 20 min at 450 rpm. CaSO_4_ was used as a cross-linker, and finally the cylindrical hydrogel composite specimens are obtained, measuring around 10 mm in height and 39 mm in diameter, as we reported in our previous result [25].

### 2.2. Characterizations

An FEI SEM-EDS inspect S50 was used to characterize the morphology and composition of the studied composites on 0.5–0.6 mm freeze-dried samples that had previously been coated with gold using the SC7620 Mini Sputter Coater/Glow Discharge System. A compressive test using the Universal Testing Machine Hung ta HT 2402 Seri 4035 was performed on a cylindrical-shaped specimen with a diameter of about 39 mm and a height of about 10 mm to evaluate the compressive strength of hydrogel. Hydrolytic degradation was carried out on 15 × 1015 × 10 mm^3^ films immersed in a flask containing 6 mL of Phosphate-Buffered Saline (PBS) with a pH of 7.0 at 37 °C. The specimens were removed from the PBS, filtered from the solution, and weighed after immersion at specific immersion periods (7, 14, 21, and 28 days). The weight of the scaffolds was measured once a week. PBS solution was added weekly after considering and ensuring all samples were completely submerged. The loss of water from the tested samples was determined on the basis of an analysis of the change in sample weight after drying for 1 h in a dryer while maintaining a constant temperature of 37. The degradation process of the Alginate/PVA/r-GO hydrogel composite was then calculated from the weight ratio at different times periodically using Equation (1), where ma is the measured mass after PBS immersion and mb is the mass before immersion.

W = ((ma − mb))/mb
(1)

The biocompatibility of the hydrogel composite was evaluated through in vitro cytotoxicity. Negative control is the live cell, and positive control is the dead cell. All samples were cut into 6 × 16 mm^2^ films with a height of 0.5 mm and sterilized using UV. Sterilized samples were immersed in 10 mL of Dulbecco’s Modified Eagle Medium (DMEM) and incubated with fibroblast baby hamster kidney 21 for 24 h at 37 °C. The Center for Veterinary Farma Surabaya performed an MTT Assay test by using fibroblast baby hamster kidney cells. The cell viability was measured based on the reduction of the [3-(4,5-dimethylthiazol-2-yl)-2,5-diphenyltetrazolium bromide] (MTT) compound to formazan using water-soluble tetrazolium dye. In this case, purple formazan crystals were formed, which indicates the presence of cell activity. The formazan product was then analyzed using a UV/Vis spectrometer (Ultrospec 1100 Pro) at 570 nm, where the OD (Optical Density) will be obtained from each well that is read. Cell viability can be calculated by comparing the OD of the sample with the OD of the control, which has no sample. The formula for finding cell viability is in Equation (2).

(2) $$Cell\;Viability=\left(\frac{\;OD\;Sampel-OD\;Sample\;control}{OD\;cell\;control-OD\;medium\;control}\right)\times 100\;\%$$

## 3. Results and Discussion

**Figure 1:**
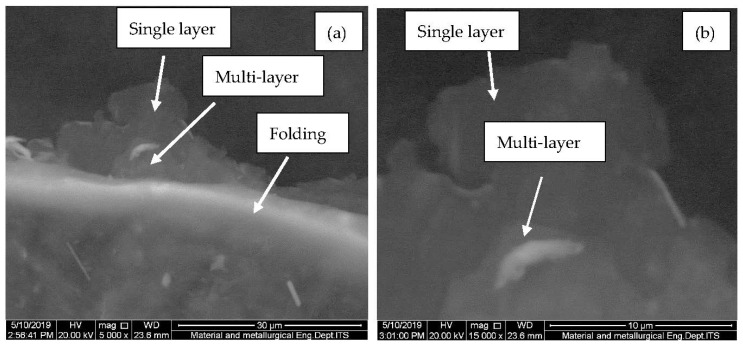
SEM image of r-GO, 5000 magnifications (**a**) and 15,000 magnifications (**b**).

The highest 2θ peak of r-GO was obtained at 24.67° based on our previous results [25,26]. At 26.88°, a graphite peak with distinctive properties is found. This finding demonstrates that when oxygen functional groups are incorporated into graphite, the interlayer spacing increases and the peak shifts, but when those functional groups are removed, the interlayer spacing lowers and the peak shifts to 24.70° in the case of the r-GO morphological structure. Figure 1 shows the transparent thin sheet consisting of a single layer, multilayers, and folding in the case of the r-GO morphological structure. This result is in accordance based on previous research by Nurdiansah et al. [25,26]. A single layer of r-GO can be observed in Figure 1, where multiple layers and folding also can occur. Indeed, the SEM image in Figure 1 confirms that the r-GO can be successfully synthesized by using Hummer’s method. 

**Figure 2:**
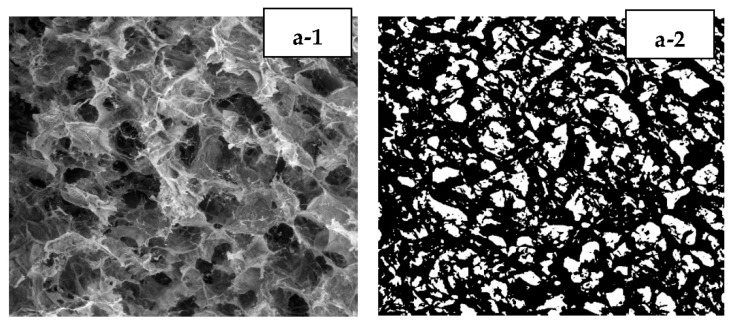

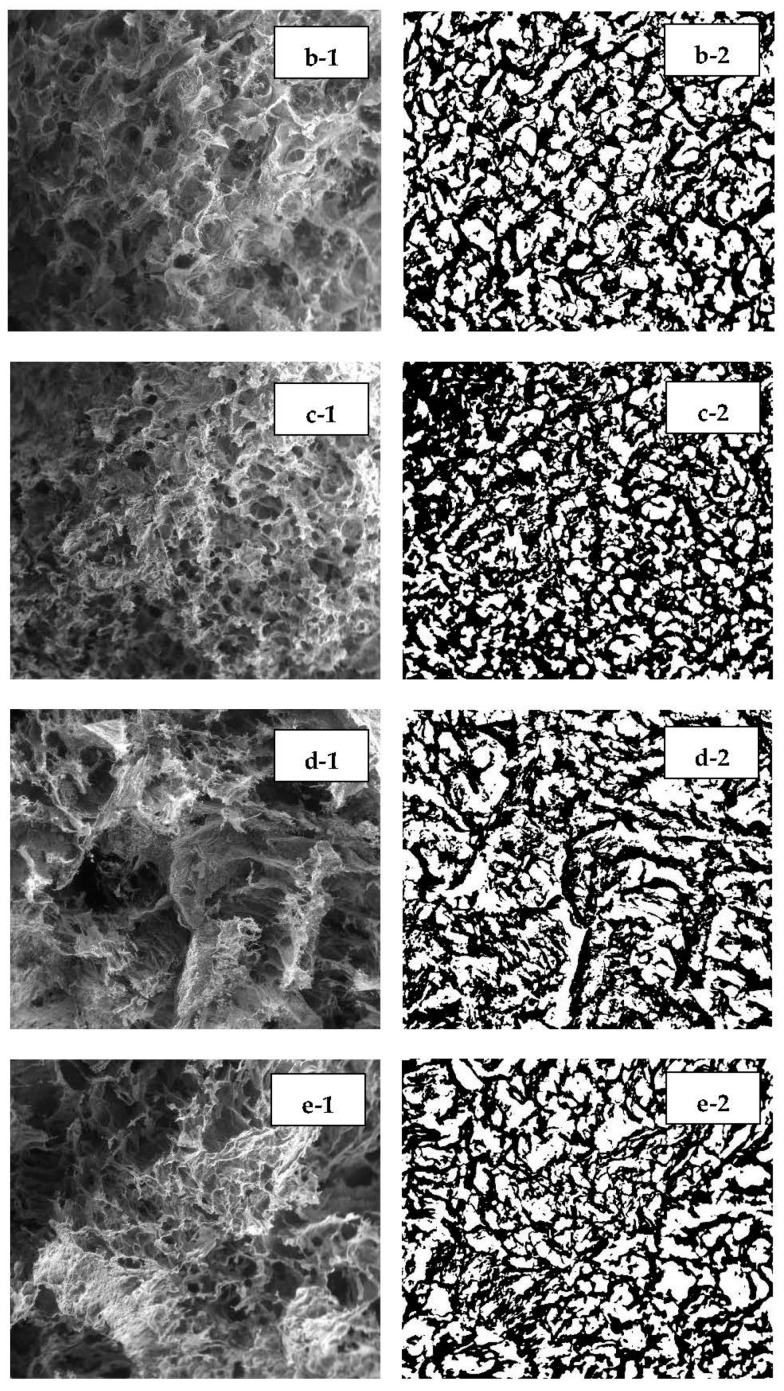
(**1**) SEM images of hydrogel Alginate/PVA/r-GO with 0 wt.% r-GO (**a-1**,**a-2**), 0.4 wt.% r-GO (**b-1**,**b-2**), 0.8 wt.% r-GO (**c-1**,**c-2**), 1.2 wt.% r-GO (**d-1**,**d-2**), and 1.6 wt.% r-GO with magnification 100× (**e-1**,**e-2**); (**2**) Results of pore identification using ImageJ software with the same composition.

**Figure 3:**
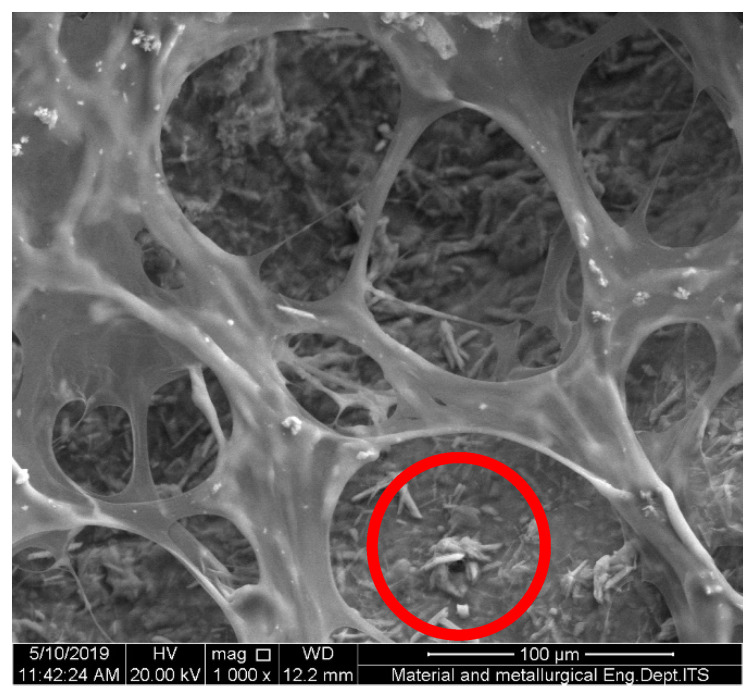
SEM images of hydrogel Alginate/PVA/r-GO with 1.6 wt.% r-GO with magnification 1000×. The microscale graphite is shown by the red circle.

Figure 2 shows the SEM images of the hydrogel Alginate/PVA/r-GO with (a) 0.4 wt.% r-GO (b), 0.8% r-GO (c), 1.2% r-GO (d), and 1.6% r-GO (e). Generally, an increase in wt.% r-GO has no significant effect on the morphology of the composites. However, the presence of some nano-sized r-GO causes agglomeration and leads to a microscale graphite form that prevents cross-linking, as shown in Figure 3 [14]. 

**Figure 4:**
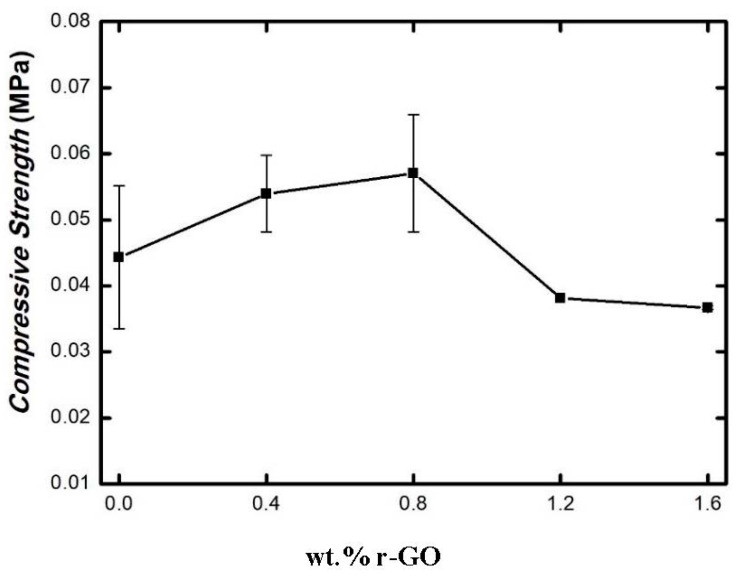
Compressive Strength vs. wt.% r-GO of the Studied Composites.

Figure 4 shows the compressive strength vs. wt.% r-GO of the studied composites. The addition of r-GO from 0.4 to 0.8 wt.% resulted in an increase in the compressive strength from 0.054 to 0.0571 MPa, respectively. In this case, the addition of 0.8 wt.% r-GO achieved the highest compressive strength. However, a decrease in the compressive strength was achieved from 0.0381 to 0.0367 MPa when the content of r-GO increased from 1.2 to 1.6 wt.%, respectively. 

Indeed, the addition of a small amount of r-GO resulted in an improvement in the mechanical properties of the composites owing to the change in crystallinity, which was triggered by the specific interaction between hydrogen bonds and the high interfacial adhesion between alginate and r-GO [10]. On the other hand, a re-stacking of the graphene occurs, preventing it from evenly dispersing in the matrix when the wt.% r-GO is too high, as shown in Figure 3. A decrease in contact surface between the matrix and the graphene resulted in a reduction in the hydrogen bond strength between the graphene and the matrix. Thus, the mechanical performance became weak. Furthermore, the presence of a gap between the graphene and the matrix caused the weak area in the composites [10,27,28]. 

**Figure 5:**
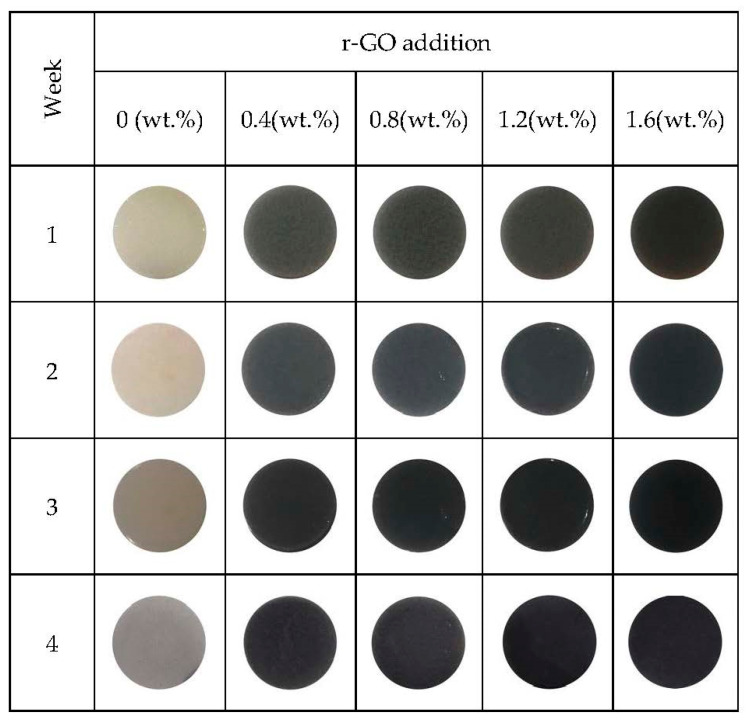
Visual observation of Alginate/PVA/r-GO for four weeks.

Figure 5 shows the visual observation of Alginate/PVA/r-GO for four weeks to determine the degradation properties. All the composites experienced no change in shape during the observation, confirming that low degradation was achieved. There is no considerable change in weight for the composites in Table 1.

Alginate is reported to be a natural biomaterial with a low degradation rate compared to other natural biomaterials regarding degradation properties [29,30]. Basically, the human body and other mammals do not have certain enzymes to digest alginate. Indeed, the ability of alginate to be degraded in the body in terms of the number of ions contained in it is poor, especially calcium ions [14,31]. Alginate contains other chemical elements, such as sodium (Na), calcium (Ca), phosphorus (P), and iron (Fe). Therefore, less pure alginates are more likely to be degraded since they contain more ions [31,32].

**Figure 6:**
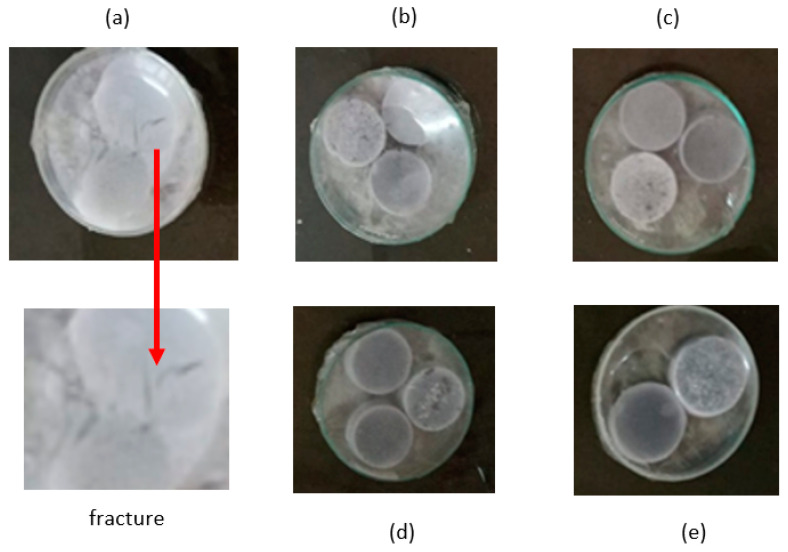
Visual observation of Alginate/PVA/r-GO at the fifth week with 0 wt.% r-GO (**a**), 0.4 wt.% r-GO (**b**), 0.8 wt.% r-GO (**c**), 1.2 wt.% r-GO (**d**), and 1.6 wt.% r-GO (**e**). The arrow shows the zoom in of the fracture area.

The degradation of Alginate/PVA/r-GO hydrogels was caused by the dissociation of Ca^2+^ ions, which are cross-linkers in alginate composites. Ca^2+^ reacts with phosphate ions in the phosphate buffer salt solution and forms calcium phosphate (CaHPO_4_), which produces a cloudy phosphate buffer solution and the hydrogel material [33]. Indeed, the degradation process of the composites is invisible. Nevertheless, a reduction in cross-links occurred, causing the material to become more fragile (as shown in Figure 6).

The degradation ratio of the studied composites is presented in Table 1. After the fourth week, the sample without r-GO addition had a higher ratio value, up to 0.432 ± 0.016, while the sample with a variation of r-GO had a lower ratio. In general, the presence of r-GO can delay the degradation of the composites. Nonetheless, no linear effect was observed between the addition of r-GO and the composite degradation ratio since, based on reports, alginate degradation happens slowly and unpredictably. In addition, the sensitivity of alginates to having cross-linked ions such as calcium, sodium, and phosphate in their environment contributes to determining the uncertainty of the degradation properties of alginates [29]. In this regard, PBS containing ions such as NaCl and Na_2_HPO_4_ can stimulate gel re-formation in alginate in the presence of the media in this study.

The weight loss and weight loss ratio data for each test condition were collected. Multiple group comparisons were performed using repeated measure analysis of variance (ANOVA) after determining significant intergroup differences by ANOVA using Statistical Package for the Social Sciences (SPSS) software. Differences with Sig < 0.05 were considered significant, whereas Sig > 0.05 indicated the same value as the control groups (0 wt.% r-GO). 

The effect of the week factor and the interaction between the week and the wt.% r-GO factor toward weight loss were analyzed using Tests of Within-Subjects. From the results of Mauchly’s Test of Sphericity, corrected with Greenhouse–Geisser correction, and the Tests of Within-Subject effects (univariate result), it is known that the value for the Greenhouse–Geisser correction on the week factor shows a sig value of 0.000, which is below 0.05 (level of significance), so the conclusion is to reject Ho. There is a significant effect from the variation of the week to the value of weight loss. In addition, the interaction factor between weeks and the wt.% addition of r-GO shows a sig value of 0.002, which is below 0.05 (level of significance), so the conclusion is reject Ho. There is also a significant effect of the interaction of week variation and wt.% addition of r-GO on the value of the weight loss.

Moreover, the effect of the week factor and the interaction between the week and the wt.% r-GO factor toward weight loss ratio were also performed. From the results of Mauchly’s Test of Sphericity, corrected with Greenhouse–Geisser correction, and the Test of Within-Subject effects (univariate result), it is known that the value for the Greenhouse–Geisser correction on the week factor shows a sig value of 0.000, which is below 0.05 (level of significance), so the conclusion is reject to Ho. There is a significant effect from the variation of the week on the value of the ratio of weight loss. In addition, the interaction factor between weeks and the wt.% addition of r-GO shows a sig value of 0.000, which is below 0.05 (level of significance), so the conclusion is to reject Ho. There is also a significant effect of the interaction of week variation and wt.% addition of r-GO on the value of the weight loss ratio.

Comparison of the weight loss in the Alginate/PVA/r-GO composite (variation of wt.% r-GO) with Control were also conducted. The results of the multiple comparisons using the Tukey HSD Post Hoc Test showed that the sample of wt.% r-GO 1.2% had significantly different (Sig < 0.05) weight loss from the control group, whereas other samples were closest to the control group (Sig > 0.05).

Furthermore, the comparison of the weight loss ratio in the Alginate/PVA/r-GO composite (variation of wt.% r-GO) with Control is investigated. The results of the multiple comparisons using the Tukey HSD Post Hoc Test showed that the samples of 0.4 wt.% r-GO, 0.8 wt.% r-GO, and 1.2 wt.% r-GO had significantly different (Sig < 0.05) weight loss ratios from the control group, whereas the 1.6 wt.% r-GO sample was closest to the control group (Sig > 0.05).

Table 2 presents degradation in the percentage of hydrogel composite Alginate/PVA/r-GO. Until the fourth week, the percentage of sample degradation with variations in the addition of r-GO ranged from 19–25%, while without the addition of r-GO, it had a higher value of 30%. Tests of Within-Subjects effects to analyze the effect of the week factor and the interaction between the week factor and the wt.% r-GO factor toward degradation in percentage are also conducted. From the results of Mauchly’s Test of Sphericity, corrected with Greenhouse–Geisser correction, and the test of within-subject effects (univariate result), it is known that the value for the Greenhouse–Geisser correction on the week factor shows a sig value of 0.000, which is below 0.05 (level of significance), so the conclusion is to reject Ho. There is a significant effect from the variation of the week to the value of the degradation in percentage. In addition, the interaction factor between weeks and the wt.% addition of r-GO shows a sig value of 0.000, which is below 0.05 (level of significance), so the conclusion is to reject Ho. There is also a significant effect of the interaction of week variation and wt.% addition of r-GO on the value of the degradation in percentage.

The comparison of the degradation in percentage in the Alginate/PVA/r-GO composite (variation of wt.% r-GO) with the Control was also conducted using the Tukey HSD Post Hoc Test. The results showed that the sample of 0.8 wt.% r-GO and 1.2 wt.% r-GO had significantly different (Sig < 0.05) weight loss ratios from the control group, whereas other samples were closest to the control group (Sig > 0.05).

**Figure 7:**
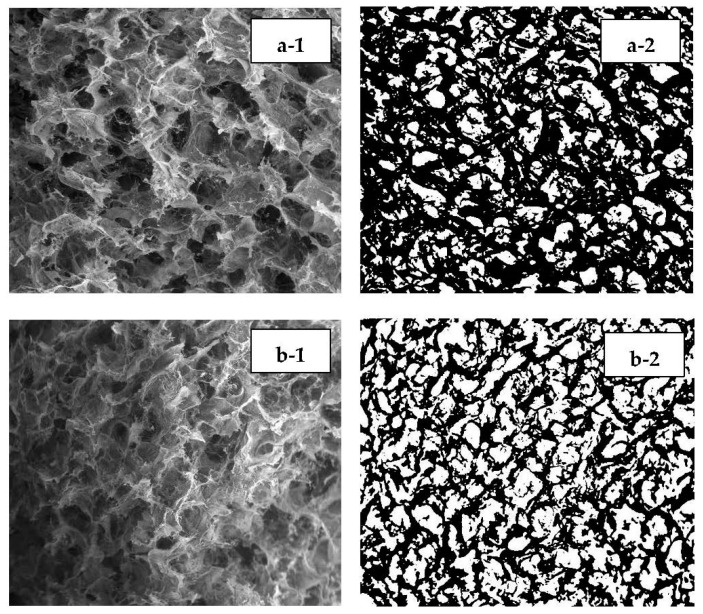

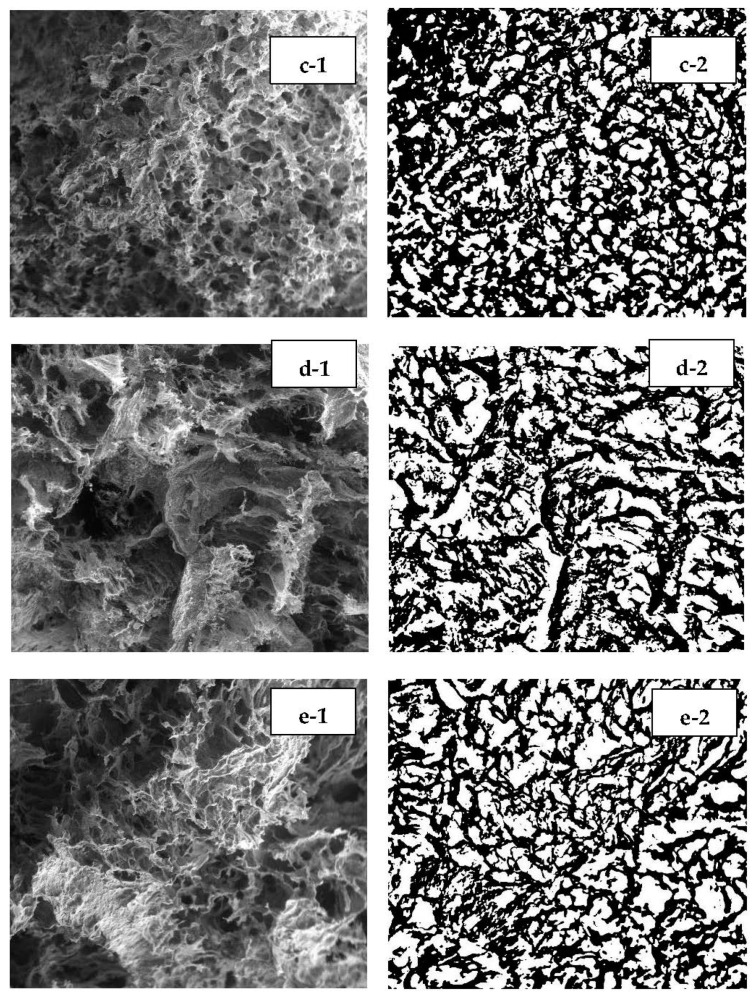
Morphological pattern of hydrogel Alginate/PVA/r-GO after degradation test for 28 days with the addition of 0 wt.% r-GO (**a-1**,**a-2**), 0.4 wt.% r-GO (**b-1**,**b-2**), 0.8 wt.% r-GO (**c-1**,**c-2**), 1.2 wt.% r-GO (**d-1**,**d-2**), and 1.6 wt.% r-GO with 100× magnifications (**e-1**,**e-2**); (**2**) indicate the results of pore identification using ImageJ software with the same composition. (1) refers to the original SEM picture (2) represent image J result.

Figure 7 shows the morphological pattern of hydrogel Alginate/PVA/r-GO after a 28-day degradation test with the addition of 0 wt.% r-GO (a), 0.4 wt.% r-GO (b), 0.8 wt.% r-GO (c), 1.2 wt.% r-GO (d), and 1.6 wt.% r-GO (e) at 500 magnifications. A significant amount of damage to the hydrogel surface due to shrinkage on the edge of the composite porosity was seen in the cross-sectional area after degradation. However, there were slight differences in the mean pore size before and after degradation of around 5–16 µm (Table 3), whereas the sample without r-GO addition had a bigger difference with 23 µm.

**Figure 8:**
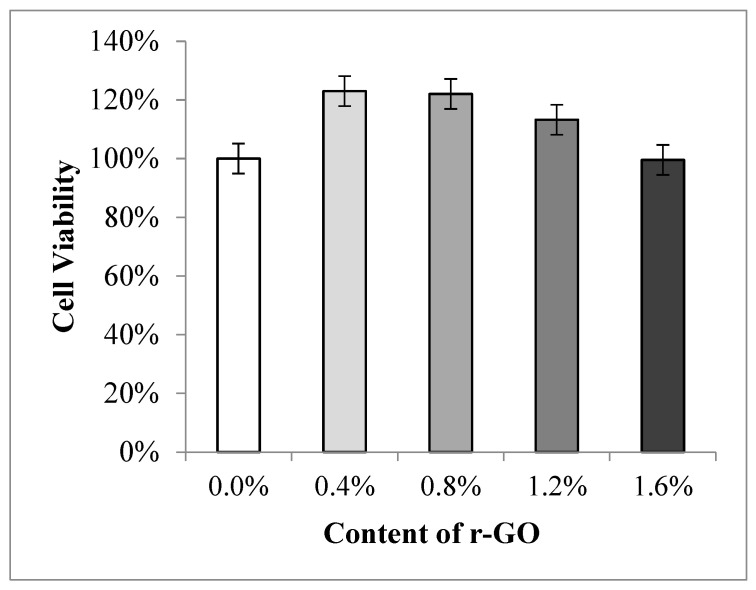
Cell Viability vs. Content of r-GO of Hydrogel Alginate/PVA/r-GO.

The cell viability vs. r-GO content of the studied composites is shown in Figure 8. The highest percentage of cell viability is shown by Alginate/PVA composites with the addition of 0.4 wt.% r-GO. The presence of r-GO in the composites had no significant effect on cell viability, as can be seen in Figure 8, where cell viability reached 100% when 0 wt.% r-GO was added. In fact, good biocompatibility of the composites with the mammalian body was successfully achieved by alginate [7]. Likewise, PVA, alginate, and PVA have no toxicity to mammalian cells [34].

Further to Figure 8, the cell viability percentage decreased with the increase of the r-GO concentration. The cell viability decreased even lower than when the r-GO was 0 wt.% when the content r-GO reached 1.6 wt.%. In high concentrations, the r-GO can cause cells to experience oxidative stress, which results in cell damage [35,36,37,38] and at low doses can induce cells to perform apoptosis, while at high doses, necrosis can occur. This behavior applies to all aerobic organisms, including aerobic bacteria. Several studies have reported that the presence of r-GO contributes to increased cell viability. In contrast, others revealed the cytotoxicity of this material, which concerned the cells used in this study and the type of assay used [39]. 

The Alginate/PVA composite with a 0.4% rGO (wt%) addition had the highest percentage of cell viability, with a value of 122.26 ± 0.93 (Table 4). The Alginate/PVA Hydrogel composite exhibits high cell survival with or without the addition of rGO, which proves that alginate is well-tolerated by mammals. These two biomaterials (alginate and PVA), which are materials, are not hazardous to mammalian cells [34].

Moreover, the addition of r-GO has also been reported to increase biocompatibility. Its rough topography can affect the surface wettability of r-GO, which in turn affects cell proliferation, although r-GO is naturally hydrophobic [40]. Therefore, it can be concluded that the addition of r-GO to the Alginate/PVA composite increased the cell viability value and decreased with the addition of the r-GO concentration [35,38].

## 4. Conclusions

Several hydrogel composites were successfully prepared by synthesizing r-GO using Hummer’s method and then by using CaSO_4_ as a crosslinker in the composite preparation with a variation in r-GO percentage. A preliminary cytotoxicity test using the MTT assay revealed that all hydrogel composites show good biological safety and non-toxicity. The addition of 0.4 wt.% r-GO presents the highest cell viability. The higher percentage of r-GO can cause some dead cells due to necrosis. Furthermore, hydrolytic degradation offers a slow degradation rate, which makes this possible for scaffolding. This good biocompatibility level and slow pace of degradation make this hydrogel composite attractive for tissue engineering applications.

## Figures and Tables

**Table 1:** Degradation ratio of hydrogel composite Alginate/PVA/r-GO *.

Sample (wt.% r-GO)	Week1	Week2	Week3	Week4
0	0.045 ± 0.007	0.171 ± 0.006	0.283 ± 0.006	0.432 ± 0.016
0.4	0.054 ± 0.013	0.098 ± 0.075	0.141 ± 0.073	0.297 ± 0.016
0.8	0.017 ± 0.008	0.041 ± 0.016	0.089 ± 0.020	0.281 ± 0.018
1.2	0.045 ± 0.018	0.080 ± 0.030	0.114 ± 0.033	0.247 ± 0.015
1.6	0.099 ± 0.041	0.165 ± 0.019	0.237 ± 0.006	0.336 ± 0.008

* The ratio is calculated from their weight at specified week divided by the initial weight.

**Table 2:** Degradation in percentage of hydrogel composite Alginate/PVA/r-GO.

Sample (wt.% r-GO)	Week1	Week2	Week3	Week4
0	4.49 ± 0.007	17.08 ± 0.006	28.28 ± 0.006	30.16 ± 0.007
0.4	5.35 ± 0.049	9.83 ± 0.075	14.07 ± 0.073	22.87 ± 0.008
0.8	1.68 ± 0.008	4.12 ± 0.017	8.95 ± 0.021	21.91 ± 0.010
1.2	3.73 ± 0.026	7.23 ± 0.042	10.63 ± 0.047	19.15 ± 0.014
1.6	10.00 ± 0.040	16.48 ± 0.019	23.67 ± 0.005	25.14 ± 0.004

**Table 3:** The comparison means pore size of hydrogel Alginate/PVA/r-GO before and after 28 days of immersion.

Sample (wt.% r-GO)	Before Degradation (µm)	After Degradation (µm)
0	2440.191 ± 3.121	2417.098 ± 3.117
0.4	5571.200 ± 3.109	5587.371 ± 3.109
0.8	3768.973 ± 2.814	3777.838 ± 2.854
1.2	3378.106 ± 2.325	3383.871 ± 2.330
1.6	4722.698 ± 2.757	4732.647 ± 2.760

**Table 4:** Value in number of Cell Viability vs. Content of r-GO of Hydrogel Alginate/PVA/r-GO.

Sample (wt.% r-GO)	Cell Viability
0	93.54 ± 1.05
0.4	122.26 ± 0.93
0.8	120.50 ± 1.44
1.2	113.21 ± 0.52
1.6	98.65 ± 0.84

## Data Availability

The data presented in this study are available on request from the corresponding author.
